# Atypical Reversible Cerebral Vasoconstriction Syndrome Without Thunderclap Headache: The Crucial Role of Medical History in Its Diagnosis and Management

**DOI:** 10.7759/cureus.87225

**Published:** 2025-07-03

**Authors:** Rei Ando, Jun Sawada, Jun Soma, Shiori Takeguchi-Kikuchi, Naoki Nakagawa

**Affiliations:** 1 Division of Respiratory Medicine and Neurology, Department of Internal Medicine, Asahikawa Medical University, Asahikawa, JPN; 2 Division of Cardiology, Nephrology, Pulmonology, and Neurology, Department of Internal Medicine, Asahikawa Medical University, Asahikawa, JPN

**Keywords:** medical history, migraine, mri, reversible cerebral vasoconstriction syndrome (rcvs), thunder clap headache

## Abstract

Reversible cerebral vasoconstriction syndrome (RCVS) is a secondary headache disorder characterized by reversible intracranial vasoconstriction. Although thunderclap headache (TCH) is a key feature of RCVS, it does not occur in all cases. We herein describe a case of a 49-year-old female patient with a history of migraine who developed RCVS. Her headache was persistent, throbbing, and located in the occipital region. It was not TCH-like, which was consistently triggered by sexual activity, and was non-responsive to non-steroidal anti-inflammatory agents (NSAIDs). Brain magnetic resonance imaging was unremarkable. Magnetic resonance angiography revealed the characteristic “strings and beads” appearance in the bilateral middle and posterior cerebral arteries and basilar artery. Therefore, she was diagnosed with RCVS. After avoiding headache triggers, including NSAIDs and sexual activity, symptoms improved within two months, with the complete resolution of vascular abnormalities and no recurrence during the follow-up period. Cases of RCVS without typical TCH are more likely to develop complications, such as coma due to stroke; however, our patient had mild symptoms and no abnormal findings in the brain parenchyma on brain MRI, which is rare. It is crucial to identify characteristic triggers, such as sexual activity, through a detailed medical history. Since RCVS and migraine share some clinical features, they may be misdiagnosed as one another. The treatment of RCVS with inappropriate drugs, including NSAIDs, triptans, and serotonin selective reuptake inhibitors, may worsen symptoms. Clinicians need to consider RCVS when patients with migraine complain of an “unusual headache” or exhibit headaches that do not respond to their usual medications. A thorough medical history and assessment of headache triggers are essential for the accurate diagnosis of RCVS, including a differential diagnosis of headache due to migraine, and the prompt identification and elimination of triggers may be the most important aspect of its management because there is currently no evidence-based medication for RCVS.

## Introduction

Reversible cerebral vasoconstriction syndrome (RCVS) is a secondary headache caused by cerebrovascular dysregulation due to symptomatic hyperactivity and is characterized by severe headache and diffuse segmental intracranial constriction that resolves within three months [1,2]. Approximately 30% of cases are accompanied by some form of stroke [3]. Therefore, it is important to diagnose RCVS correctly and provide appropriate management in outpatient settings. The typical characteristic of RCVS is thunderclap headache (TCH) [4], a sudden severe headache reaching its maximal intensity in less than one minute. Since less than 30% of patients with RCVS present with subacute or less severe headaches [5,6], the absence of typical TCH does not exclude the diagnosis of RCVS.

It has been reported that taking a medical history accounts for 78.58% of medical diagnoses [7], and this is important for headache diagnoses [8]. It is particularly useful for distinguishing between primary and secondary headaches, and the use of red flags enables the screening of dangerous secondary headaches.

We herein present a case of RCVS with a history of migraine without typical TCH. A detailed medical history and assessment of headache triggers are essential for the accurate diagnosis and effective management of RCV, and the prompt elimination of triggers may be the most important aspect of its management.

## Case presentation

A 49-year-old woman with recurrent headaches during and after sexual activity visited our outpatient department. The patient had a history of migraine with aura, with throbbing headaches over the whole head that were sometimes accompanied by nausea. Headaches typically lasted between six hours and two days and were aggravated by light and sound. She began experiencing recurring headaches with different characteristics from her usual headaches one week prior to her visit. Headaches were persistent, throbbing, and sharp in the occipital region without laterality and were always triggered by sexual activity. They gradually began during sexual activity and intensified as sexual arousal increased. The pain continued to worsen after intercourse and peaked approximately 30 minutes later. Headaches were not exacerbated by other factors, including body position. She started taking over-the-counter medication (ibuprofen 150 mg) and was prescribed diclofenac at a medical institution shortly after her symptoms began; however, neither was effective. She had cervical spondylosis in addition to migraine and took afloqualone (60 mg), diclofenac sodium (75 mg), and rebamipide (300 mg) daily. She had no history of allergies, was a social drinker and a non-smoker, and worked as a hospital receptionist. She regularly took iron supplements.

**Figure 1:**
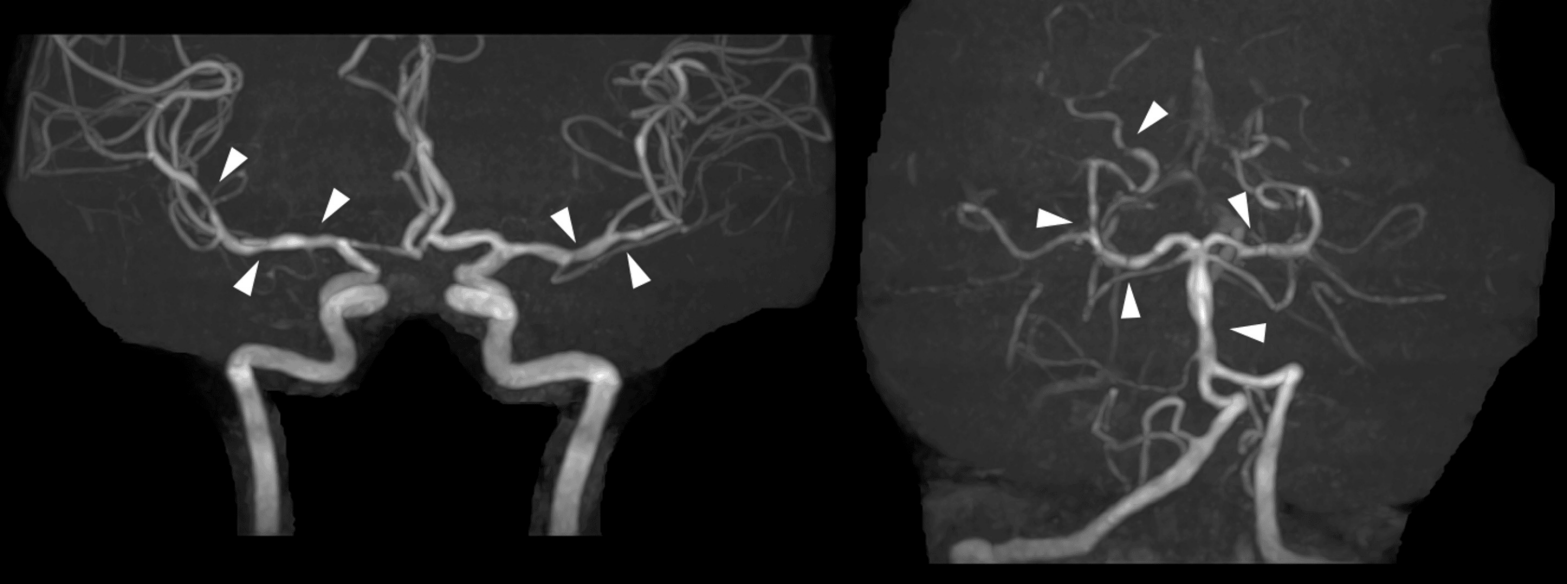
Brain magnetic resonance angiography at the initial visit to our hospital showing stenosis and dilation of bilateral middle cerebral arteries, posterior cerebral arteries, and the basilar artery (arrowhead).

A physical examination revealed no abnormalities. In a neurological examination, consciousness was alert, and there were no abnormal findings on the cranial nerves, motor and sensory nervous systems, or tendon reflexes of the limbs, and pathological reflexes, ataxia, meningeal irritation sign, and autonomic dysfunction were not present. Blood tests, including peripheral blood counts, liver and renal functions, and lipid, glucose, and electrolyte levels, were within normal ranges. Brain magnetic resonance imaging (MRI) showed no obvious abnormalities, such as deep and subcortical white matter hyperintensities in the brain parenchyma. Brain magnetic resonance angiography (MRA) revealed stenosis and dilation of the bilateral middle cerebral arteries, posterior cerebral arteries, and basilar artery, the so-called ‘strings and beads appearance’ (Figure 1). Contrast-enhanced brain MRI was not performed.

**Figure 2:**
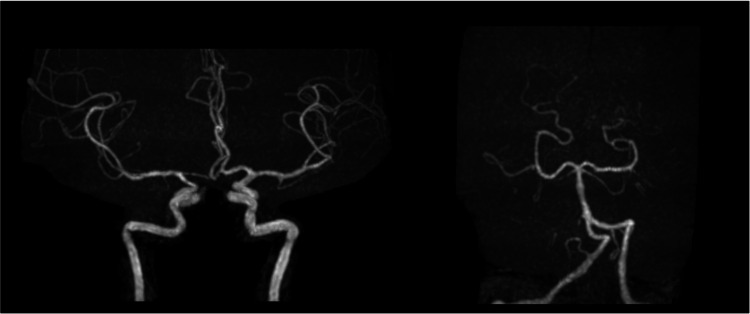
Brain magnetic angiography at two months after the initial visit to our hospital: the stenosis and dilation of cerebral arteries observed in the initial visit had disappeared.

Based on the new onset of headaches triggered by sexual activity and brain imaging findings, the patient was diagnosed with RCVS. We recommended the cessation of NSAIDs and sexual activity including intercourse, both of which appeared to trigger or worsen her symptoms. One month after her initial visit, her headaches improved but still persisted. Two months later, headaches, except for migraines, had disappeared, and vasoconstriction and dilation observed on brain MRA also disappeared (Figure 2). Brain MRI revealed no obvious abnormalities in the brain parenchyma, similar to the first visit. She subsequently did not experience any headaches triggered by the aforementioned factors, and there was no recurrence of symptoms during the follow-up period.

## Discussion

We learned the following lessons about headache management in clinical practice from the present case. Patients with RCVS may not present with TCH. Furthermore, in these cases, obtaining information on the characteristic triggers of RCVS, such as sexual activity, may lead to a correct diagnosis. 

In the ICHD-3 [9], headache caused by RCVS is described as follows: A) any new headache fulfilling criterion C, B) RCVS has been diagnosed, C) evidence of causation demonstrated by either or both of the following: 1) headache with or without a focal deficit and/or seizures has led to angiography (with the “string of beads” appearance) and the diagnosis of RCVS, 2) headache has one or more of the following characteristics: a) thunderclap onset, b) triggered by sexual activity, exertion, Valsalva maneuvers, emotion, bathing and/or showering, c) present or recurrent during ≦1 month after its onset, with no new significant headache after > 1 month, D) either of the following: 1) headache has resolved within three months of its onset and 2) headache has not yet resolved but three months from onset have not yet passed, and E) not better accounted for by another ICHD-3 diagnosis. Based on these criteria, even cases without typical TCH, such as our patient, may meet the diagnostic criteria for RCVS. RCVS has a broad clinical spectrum, and several varieties other than TCH have been described as follows: intensity may be mild, moderate, or severe, and pain may be diffuse or located in the occipital, frontal, fronto-temporal, temporal, or vertex area. Headache durations vary, lasting between several minutes and several days. Headaches may be triggered by sexual activity or diving into cold water. Although a single attack is possible, some patients have recurrent headaches [6]. Therefore, the absence of typical TCH does not exclude the diagnosis of RCVS.

A previous study reported that 30-80% of patients with RCVS may present with abnormal imaging findings in a brain imaging examination, such as subarachnoid hemorrhage, intracerebral hemorrhage, cerebral infarction, and reversible brain edema [9]. The literature described cases of RCVS without typical TCH, which were more likely to develop severe complications (i.e., coma or confusion due to stroke) [6]. Our patient had mild symptoms and no abnormal findings in the brain parenchyma on brain MRI, which made it challenging to correctly diagnose RCVS. In previous studies, the absence of TCH was observed in 0-30% of RCVS cases [4,5]. However, the majority of these cases presented with impaired consciousness due to stroke or posterior reversible encephalopathy syndrome, which may have prevented patients from complaining of headache [4,5]. It is important to note that our patient had remained alert and had not exhibited TCH throughout the disease period.

RCVS has specific triggers, the identification of which is crucial for its diagnosis [10,11]. Previous studies showed that the triggers of symptoms were identified in approximately 60-80% of RCVS cases [11,12]. Potential triggers include serotonergic agents (such as serotonin selective reuptake inhibitors (SSRI), monoamine oxidase inhibitors, and triptans), vasoactive substances (including sympathomimetic agents, caffeine, tobacco, NSAIDs, and stimulants), peripartum and postpartum periods [12], and excessive physical stress (such as marathon running, sexual activity, and cold exposure) [13]. The most common trigger was vasoactive substances, accounting for 41.4% of cases [11]. Other major triggers included pregnancy and the postpartum period (20.9%) and sexual activity (10.5%) [11]. A thorough medical history to identify potential triggers facilitates the diagnosis of RCVS, enabling appropriate brain imaging studies and subsequent clinical management, even in patients with an atypical presentation such as the present case.

In our case, sexual intercourse was identified as a trigger of headache through the patient’s medical history. Sexual orgasm may act as a vasoconstrictive trigger; therefore, sexual activity is one of the important triggers of RCVS [14]. Headache associated with sexual activity (HSA) is classified as primary headache associated with sexual activity (PHASA) in ICHD-3 [9]. The clinical features of PHASA closely resemble and often overlap with secondary causes that may also trigger HAS, such as RCVS, subarachnoid hemorrhage, arterial dissection, and venous abnormalities [14]. Therefore, secondary causes have to be ruled out in order to correctly diagnose PHASA. In previous studies [14,15], 67-84.5% of HAS cases were caused by secondary headaches, the majority of which were due to RCVS. A radiological assessment was shown to successfully classify repeated HSA [14]. Therefore, a brain imaging examination needs to be performed in order to identify cerebrovascular disorders among patients with HAS and ensure a proper diagnosis and treatment.

Randomized clinical trials have yet to be conducted on treatments for RCVS [11]. A literature review showed that calcium channel blockers were used to treat approximately 80% of patients with RCVS, and prospective or retrospective studies indicated that they attenuated the symptoms of RCVS; however, the beneficial effects of calcium channel blockers on cerebral vasoconstriction remain unclear [11]. On the other hand, another study demonstrated that with an appropriate diagnosis and management, RCVS typically resolved within one month from its onset, with the disappearance of severe headaches and other clinical manifestations [16]. Moreover, cerebral vasoconstriction significantly improved or disappeared completely within three months. Therefore, the prompt identification and elimination of triggers of RCVS may be the most important aspect of its management because there is currently no evidence-based medication for RCVS [11].

As demonstrated in the present case, the management of RCVS is challenging in patients with a history of migraine. Migraine is a common preexisting condition in RCVS patients, with a prevalence of 17-57% [12,17,18]. Since both conditions share some clinical features, they may be misdiagnosed as one another. If patients with RCVS are misdiagnosed with migraine, commonly used migraine medication, such as NSAIDs, triptans, and SSRIs, may be administered and worsen the condition. As an alternative treatment, acetaminophen and lasmiditan may be useful for managing migraine attacks in patients with RCVS. Lasmiditan is a serotonergic agent that selectively targets 5-HT1F receptors and lacks vasoconstrictive effects, while triptans act on 5-HT1B/1D receptors and cause vasoconstriction [19]. Headaches develop more abruptly in patients with RCVS than in those with migraines and are of a distinct duration [20]. Therefore, particular attention is needed when patients with migraine complain of an “unusual headache” that differs from their typical episodes, as well as headaches that do not respond to their usual medications because they may develop RCVS. Among cases of RCVS with a history of migraine, deep and subcortical white matter hyperintensities on brain MRI were significantly more frequent and often worsened in the chronic phase than in cases of RCVS without a history of migraine [18]. Our patient had mild symptoms and no abnormal findings in the brain parenchyma on brain MRI in both the first visit and chronic phase. This may reflect the broad clinical spectrum of RCVS and migraine. Early interventions may prevent the development of brain parenchymal lesions on MRI in the chronic phase. 

## Conclusions

Although TCH is the classical and most common symptom of RCVS, its absence does not exclude a diagnosis of RCVS; therefore, it is difficult to diagnose RCVS correctly. This case highlights that even without TCH or focal deficits, a thorough clinical history, particularly the identification of known RCVS triggers such as sexual activity, can be instrumental in making an accurate diagnosis. In patients with migraine, differentiating RCVS from their usual headache pattern is particularly difficult due to overlapping features and the potential for symptom misattribution. Importantly, the use of common migraine medications such as triptans and SSRIs may exacerbate RCVS, underscoring the need for diagnostic vigilance. Headache associated with sexual activity warrants neuroimaging to exclude secondary causes such as RCVS, which accounts for a significant proportion of these cases. As demonstrated here, trigger identification guided appropriate imaging and prevented mismanagement. Ultimately, the careful assessment of headache characteristics, medication response, and potential precipitating factors plays a central role in distinguishing RCVS from other headache disorders and guiding safe and effective clinical management of RCVS, and the prompt identification and elimination of triggers may be the most important aspect of its management.
